# Emerging Biomarkers for Predicting Clinical Outcomes in Patients with Heart Disease

**DOI:** 10.3390/life13010230

**Published:** 2023-01-13

**Authors:** Krisztina Pál, Ion-Bogdan Mănescu, Silvia Lupu, Minodora Dobreanu

**Affiliations:** 1 Clinical Laboratory, County Emergency Clinical Hospital of Targu Mures, 540136 Targu Mures, Romania; 2 Department of Laboratory Medicine, George Emil Palade University of Medicine, Pharmacy, Science, and Technology of Targu Mures, 540142 Targu Mures, Romania; 3 Internal Medicine V, George Emil Palade University of Medicine, Pharmacy, Science, and Technology of Targu Mures, 540142 Targu Mures, Romania; 4 1st Department of Cardiology, Emergency Institute for Cardiovascular Disease and Heart Transplant of Targu Mures, 540136 Targu Mures, Romania; 5 Center for Advanced Medical and Pharmaceutical Research, George Emil Palade University of Medicine, Pharmacy, Science, and Technology of Targu Mures, 540139 Targu Mures, Romania

**Keywords:** coronary artery disease, acute coronary syndrome, heart failure, biomarkers

## Abstract

Cardiovascular disease is most frequently caused by the development and progression of atherosclerosis. When coronary arteries are afflicted, and the stenoses caused by atherosclerotic plaques are severe enough, the metabolic supply-and-offer balance is disturbed, leading to myocardial ischemia. If atherosclerotic plaques become unstable and local thrombosis develops, a myocardial infarction occurs. Sometimes, myocardial ischemia and infarction may result in significant and irreversible heart failure. To prevent severe complications, such as acute coronary syndromes and ischemia-related heart failure, extensive efforts have been made for developing biomarkers that would help identify patients at increased risk for cardiovascular events. In this two-part study, we attempted to provide a review of existing knowledge of blood biomarkers that may be used in this setting. The first part of this work was dedicated to conventional biomarkers, which are already used in clinical practice. In the second part, here presented, we discuss emerging biomarkers which have not yet become mainstream.

## 1. Introduction

Cardiovascular disease (CVD) is very often caused by myocardial ischemia due to the development and progression of atherosclerosis. Coronary artery disease (CAD) includes acute coronary syndromes (ACS), such as myocardial infarction (MI) and unstable angina, and chronic coronary syndromes. Coronary artery disease may result in heart failure (HF) and is associated with poor clinical outcomes. In order to better assess the risk for poor cardiovascular (CV) outcomes, many humoral parameters have been investigated over the years as potential biomarkers for prognosis, with mixed results. Although progress has been made and some parameters proved to be sufficiently reliable for this purpose, the perfect biomarker has not been found.

In this two-part work, we attempted to provide a comprehensive review of recent data on conventional and emerging biomarkers of CVD. The second part focuses on novel, emerging biomarkers. We attempted to select the most relevant parameters that may be used as biomarkers in this setting from recently published original articles. In this two-part work, we focused on summarizing existing knowledge on the value of blood markers for predicting outcomes. Consequently, the pathophysiological mechanisms behind each biomarker are only briefly explained.

## 2. Materials and Methods

For the purposes of the current work, we conducted a systematic search in the PubMed database. The following key words were used to identify relevant articles: “(myocardial ischemia) AND (heart failure) AND (prognosis OR outcome OR prognostic factor OR prediction) AND (biomarker OR marker)”. Only articles published between 2015–2022 were selected. Secondary research articles (reviews, systematic reviews, or meta-analyses) were generally excluded. Some older articles or reviews may have been cited if needed to explain the pathophysiological rationale for researching a certain molecule as a possible biomarker of CVD.

Our work is structured in two parts—the first was dedicated to validated biomarkers, already used for diagnostic and/or prognostic purposes in patients with CVD, and in the second part, here presented, we attempted to include the most relevant recent research regarding emerging biomarkers for use in patients with CVD.

Emerging biomarkers are briefly described in order to familiarize the reader with the pathophysiological mechanisms behind their use.

## 3. Results

### 3.1. Inflammatory Markers

As sustained by extensive data, inflammation plays a crucial role in the process of atherosclerosis. For this reason, there has been a lot of focus on inflammatory parameters which could be used as markers of prognosis in CVD. Among inflammation-related biomarkers, only C-Reactive Protein (CRP) has become mainstream, and, for that reason, we mentioned recent findings on this parameter in the first part of our work.

Further, we present other humoral markers of inflammation which may evolve into biomarkers for CVD, if endorsed by sufficient and solid data. Relevant statistical data in this regard have been included in Table 1.

#### 3.1.1. Serum Amyloid A

Serum amyloid A (SAA) is an acute phase protein with persistently elevated levels in chronic inflammation, which seems to be linked to atherosclerosis development and may therefore play a part in CV risk prediction [1]. Zewinger et al. investigated the association between SAA and high-density lipoprotein cholesterol (HDL-C), relying on the premise that the interaction between SAA and anti-atherogenic HDLs (high-density lipoproteins) can lead to structural alterations associated with pro-atherosclerotic effects and overall worse CV outcomes [2]. In this study, 3310 patients undergoing coronary angiography were followed over a median of 9.9 years, and SAA was shown to have predictive value for CV mortality. In addition, a formula for calculating biologically effective HDL-C, while taking SAA concentrations into account, was elaborated, which might prove to be a feasible method of estimating the biological effectiveness of HDLs compared to more laborious techniques [2].

#### 3.1.2. Obesity and Inflammation

Obesity is known to be associated with a systemic proinflammatory status by maintaining a chronic low-grade inflammation and thus contributing to atherogenesis [3]. Jia et al. explored the relationship between body weight, expressed by body mass index (BMI) and high-sensitivity C-reactive protein (hs-CRP) in an observational cohort study including 478 patients with unstable angina undergoing elective percutaneous coronary intervention (PCI) [4]. Patients meeting the composite endpoint of MACE (major adverse CV events, defined as CV death, heart failure, stroke, myocardial infarction, or repeated revascularization) had a significantly higher hs-CRP (*p* = 0.033). Furthermore, the hs-CRP/BMI ratio was independently associated with the composite endpoint on multivariate analysis. This study concludes that hs-CRP levels should be carefully interpreted and that adjusting this parameter by BMI might actually depict patients’ inflammatory status more accurately [4].

#### 3.1.3. Albumin

Systemic inflammation influences the levels of serum albumin by decreasing its rate of synthesis. Hypoalbuminemia is an expression of the inflammatory process and was previously studied for its prognostic role in patients with acute HF and/or cardiogenic shock [5,6]. Plakht et al. performed a retrospective study on 8750 acute MI survivors and reported that serum albumin levels at admission were predictive for long-term mortality (median follow-up 6.1 years) [7]. Mortality increased with decreasing serum albumin levels, from 17.6% for albumin > 4.1 g/dL to 57.5% for albumin < 3.4 g/dL (*p* for trend < 0.001). When using albumin > 4.1 g/dL as reference, the other albumin groups had a significantly increased long-term mortality risk [7].

#### 3.1.4. Markers of Iron Metabolism

Iron deficiency is a frequent comorbidity in patients with chronic HF, leading to a decreased quality of life and worse overall clinical prognosis [8]. A cohort study investigated the prognostic relevance of biomarkers of iron status after an ACS by performing high-frequency blood sampling (a median of 17 determinations) in 844 patients over a one-year follow-up period [9]. The study revealed a higher incidence of the composite outcome of CV mortality and recurrent nonfatal ACS with decreasing log-iron, as well as with decreasing log-TSAT (transferrin saturation). However, no associations with the composite outcome were recorded for ferritin and transferrin [9].

In the BIOSTAT-CHF study, which enrolled 1453 patients with worsening HF and iron deficiency, participants were divided in two different groups: low iron storage (*n* = 960) or defective iron utilization (*n* = 493), according to criteria validated in a previous, smaller study (DEFINE-HF, *n* = 42) [10]. Findings from this study revealed that low iron storage was an independent predictor of the composite outcome of all-cause mortality or HF hospitalization. Contrarily, defective iron utilization was not associated with the outcome [10].

**Table 1:** Predictive value of inflammatory markers for clinical outcomes in patients with ischemia-related cardiovascular diseases.

Biomarker	Details	Outcome(s)	Follow-Up	n	Predictor/Cut-Off Value	Risk [95% CI]	Ref.
SAA	Patients undergoing coronary angiography	All-cause mortality	9.9 years	3310	>16.90 mg/L	HR 1.58 [1.26–1.97]	[2]
		CV mortality				HR 1.72 [1.30–2.28]	
hs-CRP/BMI	Elective PCI for UA	MACE (CV death, HF, stroke, MI, or repeated revascularization)	4.2 years	478	Upper tertile vs. Lower tertile	HR 2.03 [1.17–3.50]	[4]
Serum Albumin (admission)	MI survivors	All-cause mortality	6.1 years	8750	Reference: >4.1 g/dL a. 3.9–4.1 g/dL; b. 3.7–3.9 g/dL; c. 3.4–3.7 g/dL; d. <3.4 g/dL	a. HR 1.45 [1.24–1.70]	[7]
						b. HR 1.71 [1.47–1.98]	
						c. HR 2.45 [2.14–2.81]	
						d. HR 4.33 [3.79–4.94]	
Iron metabolism	Post-ACS	CV mortality and recurrent nonfatal ACS	1 year	844	log-iron, per 1 SD decrease	HR 2.19 [1.34–3.54]	[9]
					log-TSAT, per 1 SD decrease	HR 1.78 [1.17–2.65]	
	Worsening HF and iron deficiency	All-cause mortality or HHF	1.8 years	1453	low iron storage (transferrin saturation < 20%; serum ferritin ≤ 128 ng/mL)	HR 1.47 [1.26–1.71]	[10]

Abbreviations: ACS, acute coronary syndrome; BMI, body mass index; CI, confidence interval; CV, cardiovascular; HF, heart failure; HHF, heart failure hospitalization; HR, hazard ratio; hs-CRP, high-sensitivity C-reactive protein; MACE, major adverse cardiovascular events; MI, myocardial infarction; n, number of participants; PCI, percutaneous coronary intervention; Ref, reference; SAA, serum amyloid A; SD, standard deviation; TSAT, transferrin saturation; UA, unstable angina.

### 3.2. Cytokines

Cytokines play a key role in the inflammatory cascade and are therefore considered to be heavily involved in the process of atherogenesis. A considerable number of studies aimed to evaluate the prognostic value of cytokines with regard to adverse CV outcomes. In this review, we summarize the most recent findings concerning interleukins, the soluble suppression of tumorigenesis-2, growth differentiation factor-15, leptin, and tumor necrosis factor superfamily member 14 in different clinical settings. To facilitate reading, the relevant statistical data are included in Table 2.

#### 3.2.1. Interleukin-6 (IL-6)

IL-6 is a pro-inflammatory cytokine with pleiotropic effects, linked to atherosclerosis development and plaque vulnerability. The SOLID-TIMI 52 Trial evaluated serum IL-6 concentrations in 4939 patients with a recent ACS (≤30 days) over a median follow-up period of 2.5 years [11]. Serum IL-6 values in the highest quartile were associated with a higher risk of MACE. Furthermore, IL-6 was independently associated with an increased risk of MACE and CV death or HF after adjusting for established biomarkers [11]. On the other hand, an observational cohort study of patients with STEMI and primary PCI (*n* = 989, median follow-up of 4.6 years) [12] focusing on IL-6, CRP, and components of the IL-6 signaling pathway, such as soluble IL-6 receptor (sIL-6R) and soluble glycoprotein 130 (sgp130), found no significant relationship between IL-6 or sgp130 and adverse cardiac outcomes. However, sIL-6R concentration in the upper quartile (>47.7 ng/mL) was significantly associated with an increased risk of the primary endpoint (a composite of all-cause mortality, MI, stroke, unplanned revascularization, or HF rehospitalization) [12].

Another study of 14,611 participants from the STABILITY trial focused on the predictive value of plasma IL-6 and hs-CRP concentrations in patients with stable coronary heart disease (CHD) [13]. Increased IL-6 values were significantly associated with a higher risk of MACE (composite of CV death, MI or stroke), CV death, MI, HF, and all-cause mortality [13]. On multivariate adjustment, IL-6, but not hs-CRP, was an independent predictor of the aforementioned outcomes [13].

#### 3.2.2. Interleukin-34 (IL-34)

The pro-inflammatory cytokine IL-34, a multifunctional cytokine that participates in the differentiation and proliferation of mononuclear phagocytes, was advanced as a novel biomarker of impaired renal function and was recently studied in HF patients. An observational study of 510 patients with stable HF reported the significant associations between IL-34 levels and the primary endpoint (a composite of CV death and HF hospitalization), CV death alone, HF hospitalization alone, and all-cause mortality [14]. Multivariate analysis showed the benefit of adding IL-34 to conventional risk factors for predicting the primary endpoint (AUC = 0.768 with IL-34 vs. AUC = 0.724 without IL-34). This analysis demonstrated the role of IL-34 as a predictor of poor outcomes in patients with HF, especially those who also have kidney impairment [14].

#### 3.2.3. Interleukin-12 (IL-12) and Interleukin-18 (IL-18)

Opstad et al. investigated the proposed synergic effect of the inflammatory cytokines IL-18 and IL-12 on the prognosis of stable CAD patients in an observational study of 1001 patients [15]. Statistical analysis revealed no association between CV events (primary endpoint defined as fatal/nonfatal AMI, unstable angina, ischemic stroke, or death) and values of IL-18 or IL-12 alone when comparing upper tertiles of these parameters to the lower tertile [15]. However, the simultaneous analysis of IL-18 and IL-12 showed an increased risk of CV events when both biomarkers were in the upper tertile [15].

#### 3.2.4. Soluble Suppression of Tumorigenesis-2 (sST2)

The suppression of tumorigenesis-2 (ST2) is a member of the IL-1 receptor family, which has two isoforms—a transmembrane form, which binds IL-33 and is involved in the immune response, and a soluble (sST2) form, which acts as a decoy receptor and may prevent exaggerated responses, while also preventing the beneficial effects of the ST2–IL 33 interaction [16]. Because sST2 is present in peripheral blood, it can be measured, and could be used as a marker of prognosis in patients with CVD [16].

Because of its proven role in inflammation, sST2 was tested in multiple clinical settings with the purpose of demonstrating its use as a marker of prognosis in patients with CVD. In a recent prospective cohort study of 3641 patients with CAD, followed for a median of 6.4 years, elevated sST2 levels (>19 ng/mL) were correlated with a high risk of MACEs (composite of cardiac death, MI, unstable angina, or unplanned revascularization) and all-cause mortality [17]. Moreover, in patients with diabetes mellitus, the predictive value of elevated sST2 levels for MACEs and all-cause death was even higher than in non-diabetic patients [17].

In addition to that, the prognostic value of sST2 was tested in acute settings. For instance, in a prospective study of 295 patients undergoing primary PCI for STEMI, the elevated baseline sST2 (>58.7 ng/mL) was independently associated with mortality during the 12-month follow-up period [18]. Additionally, in another small study of 379 STEMI patients, sST2 levels were significantly higher in people with no-reflow after PCI, while patients with sST2 >11.6 ng/mL had an increased risk for no-reflow, independently of other risk factors [19]. Consequently, both studies suggest that elevated sST2 measured in acute setting is associated with worse outcomes, although the reported cut-off values are rather different.

Minana et al. tried a different approach for demonstrating the prognostic use of sST2 in STEMI patients [20]. The authors measured sST2 levels 24 h after primary PCI for STEMI in 109 patients and then performed cardiac magnetic resonance imaging at 1 week and 6 months post-STEMI. Their results showed that higher sST2 was associated with a larger infarct size and less myocardial salvage within 1 week after PCI [20]. Moreover, sST2 proved to be an independent predictor of lower left ventricular ejection fraction (LVEF), higher left ventricular end-systolic volume, and infarct size [20].

By contrast, data from the BIOSTRAT study suggested that galectin-3 performs better than sST2 for predicting the primary endpoint (CV death or hospitalization) [21]. Data was collected from 117 patients with first-time STEMI treated by primary PCI and followed for 12 months; only baseline Galectin-3 (Gal-3), and not sST2, was a predictor of the primary endpoint, independently of other factors [21].

#### 3.2.5. Growth Differentiation Factor-15 (GDF-15)

Growth differentiation factor 15 (GDF-15) belongs to the transforming growth factor-β (TGF-β) superfamily, previously known as the macrophage-inhibiting cytokine, which increases as a consequence of inflammation and oxidative stress [22]. Because atherosclerosis is an inflammatory process, it has been hypothesized that GDF-15 may be used as a valid marker of prognosis in patients with atherosclerosis-related CVD. Recently, an observational cohort study evaluated the prognostic value of plasma GDF-15 in 3641 patients with CAD, who were followed up for 5.3–7.6 years [22]. In this study, elevated GDF-15 levels > 1800 ng/L were independently associated with the primary endpoint (composite of all-cause mortality, ACS, or unplanned coronary revascularization) and all-cause mortality [22]. Moreover, GDF-15 provided incremental prognostic value to a model, including clinical data (age, sex, BMI, smoking, hypertension, diabetes mellitus, and hyperlipidemia) (AUC 0.628 vs. 0.583) [22].

Similar findings were reported after a substudy of 14,577 patients diagnosed with stable CAD from the STABILITY (Stabilization of Atherosclerotic Plaque by Initiation of Darapladib Therapy) trial [23]. In this study, there was a linear relationship between increasing GDF-15 levels and the primary endpoint defined as a composite of CV death, nonfatal MI, or nonfatal stroke [23]. By comparison with people in the lowest quartile, patients in the highest quartile of GDF-15 had a significantly increased risk for the primary outcome, HF hospitalization, HF death, MI, stroke, and CV death [23]. After adjusting for clinical factors, GDF-15 was independently associated with CV mortality, non-CV mortality, and stroke, but not MI [23]. Additionally, GDF-15 proved to be a reliable predictor of adverse outcomes in the Platelet Inhibition and Patient Outcomes (PLATO) trial. In the biomarker substudy of PLATO, performed on 17,095 patients with ACS, GDF-15 was the second strongest marker for all-cause mortality prediction after NT-proBNP [24]. Baseline GDF-15 levels were associated with a three-fold increase in risk of HF death and an almost two-fold increase in risk of sudden cardiac death or arrhythmia [24]. This study also put forward the potential benefit of using GDF-15 as a predictor of death due to major bleeding in coronary heart disease [24].

#### 3.2.6. Leptin

Leptin is a protein with dual function, acting as a hormone to preserve energy homeostasis, and as a cytokine, eliciting inflammatory responses; due to its proven involvement in inflammation, it has been postulated that it may be used as a marker of prognosis in patients with CVD [25]. The most consistent data comes from a substudy of the ARTEMIS trial. According to Puurunen et al. [26], in a group of 1946 participants with stable CAD from the ARTEMIS trial, leptin had predictive value for the primary composite endpoint (MACE defined as CHF hospitalization or cardiac death) and the secondary endpoint (ACS or stroke) over a follow-up period of 2 years. Moreover, plasma leptin levels >14.1 ng/mL were independently associated with a significant increase in risk of MACE, while leptin levels > 9.9 ng/mL predicted a higher risk of ACS or stroke [26]. Additionally, the inclusion of leptin in a conventional risk model (including age, sex, BMI, LVEF, and presence of diabetes) significantly improved the prediction of MACE in patients with stable CAD (C-index 0.81 vs. 0.78) [26].

There is also data on leptin from patients with ACS. Ritsinger et al. studied the value of adipokines (plasma leptin and total adiponectin) as predictive biomarkers in 181 patients with AMI and no known diagnosis of diabetes, over a median follow-up of 11.6 years. While adiponectin at discharge (>14.4 mg/L in men and >21.7 mg/L in women) was correlated with total mortality, leptin levels showed no predictive value for total mortality or major CV events during follow-up [27].

#### 3.2.7. TNFSF14

TNFSF14 is a transmembrane protein belonging to the tumor necrosis factor (TNF) ligand family, which may have a pathogenic function in atherosclerosis. Recent data are limited. In a recent prospective multicenter study, involving 894 patients with stable CAD, elevated levels of TNFSF14 were independently associated with adverse CV outcomes (composite of CV death, nonfatal MI, and stroke) [28].

**Table 2:** Predictive value of cytokines for clinical outcomes in patients with ischemia-related cardiovascular diseases.

Biomarker(s)	Details	Outcome(s)	Follow-Up	n	Predictor/Cut-Off Value	Risk [95% CI]	Ref.
IL-6	Recent ACS	MACE (CHD death, MI or urgent revascularization)	2.5 years	4939	Q4 (>3.97 pg/mL) vs. Q1 (≤1.16 pg/mL)	HR 1.57 [1.22–2.03]	[11]
	Stable CHD	MACE (CV death, MI or stroke)	3.7 years	14,611	Q4 (≥3.2 pg/mL) vs. Q1 (<1.4 pg/mL)	HR 1.60 [1.30–1.97]	[13]
		HHF				HR 2.28 [1.34–3.89]	
		MI				HR 1.53 [1.14–2.04]	
		All-cause mortality				HR 2.11 [1.62–2.76]	
		CV death				HR 2.15 [1.53–3.04]	
sIL-6R	STEMI + PCI	All-cause mortality, MI, stroke, unplanned revascularization, or HHF	4.6 years	989	>47.7 ng/mL	HR 1.54 [1.08–2.21]	[12]
IL-34	Stable HF	CV death and HHF	2 years	510	Q4 (≥114.79 pg/mL) vs. Q1 (<54.56 pg/mL)	HR 2.20 [1.24–3.89]	[14]
		CV death			log(IL-34), per SD increase	HR 1.34 [1.09–1.65]	
		HHF				HR 1.23 [1.01–1.49]	
		All-cause mortality				HR 1.34 [1.11–1.61]	
IL-18 and IL-12	Stable CAD	Fatal/nonfatal MI, UA, ischemic stroke, or death	2 years	1001	IL-18 ≥ 293.1 pg/mL and IL-12 ≥ 115.2 pg/mL (tertile 3 vs. tertile 1 for both)	OR 1.90 [1.11–2.61]	[15]
sST2	CAD	Cardiac death, MI, UA or unplanned revascularization	6.4 years	3641	>19 ng/mL	HR 1.36 [1.17–1.56]	[17]
		All-cause mortality				HR 2.01 [1.56–2.59]	
	STEMI + PCI	Mortality	1 year	295	>58.7 ng/mL	HR 5.01 [1.02–16.30]	[18]
	STEMI	No-reflow	In-hospital	379	>11.6 ng/mL	HR 2.74 [1.43–5.24]	[19]
Gal-3	First STEMI + PCI	CV death or HHF	1 year	117	≥9.57 ng/mL	HR 8.65 [1.45–51.70]	[21]
GDF-15	CAD	All-cause mortality, ACS, or unplanned revascularization	6.4 years	3641	>1800 ng/L	HR 1.74 [1.44–2.02]	[22]
		All-cause mortality				HR 2.04 [1.57–2.61]	
	Stable CAD	CV death, nonfatal MI, or nonfatal stroke	3.7 years	14,577	Q4 (≥1827 ng/L) vs. Q1 (<915 ng/L)	HR 1.8 [1.5–2.2]	[23]
		HHF				HR 5.8 [3.2–10]	
		HF death				HR 4.3 [1.3–14]	
		MI				HR 1.4 [1.1–1.9]	
		Stroke				HR 1.8 [1.1–2.8]	
		CV death				HR 2.63 [1.9–3.6]	
	ACS	All-cause mortality	1 year	17,095	Q4 vs. Q1 of log-transformed values (median 1550 ng/L, IQR 1145–2219 ng/L)	HR 2.65 [2.17–3.24]	[24]
Leptin	Stable CAD	CHF hospitalization or cardiac death	2 years	1946	>14.1 ng/mL	HR 3.37 [1.64–6.90]	[26]
		ACS or stroke			>9.9 ng/mL	HR 1.95 [1.29–2.96]	
Adiponectin (at discharge)	AMI (no known DM)	Total mortality	11.6 years	181	>14.4 mg/L (men) and >21.7 mg/L (women)	HR 3.57 [1.30–9.82]	[27]
TNFSF14	Stable CAD	CV death, nonfatal MI, and stroke	24 months	894	Per 100 pg/mL increment	HR 1.14 [1.04–1.25]	[28]

Abbreviations: ACS, acute coronary syndrome; CAD, coronary artery disease; CHD, coronary heart disease; CHF, congestive heart failure; CI, confidence interval; CV, cardiovascular; DM, diabetes mellitus; Gal-3, galectin 3; GDF-15, growth differentiation factor-15; HF, heart failure; HHF, heart failure hospitalization; HR, hazard ratio; IL, interleukin; MACE, major adverse cardiovascular events; MI, myocardial infarction; n, number of participants; OR, odds ratio; PCI, percutaneous coronary intervention; Ref, reference; sIL-6R, soluble IL-6, receptor; sST2, soluble suppression of tumorigenesis-2; STEMI, ST segment elevation myocardial infarction; TNFSF14, tumor necrosis factor superfamily member 14; UA, unstable angina; Q, quartile.

### 3.3. Hematological Biomarkers

The pathophysiological processes behind atherosclerosis, plaque obstruction, and occurrence of ACS are manifold, including immune processes, inflammation, and therefore all major circulating blood cell types. Platelets are involved in plaque progression and occurrence of ACS. Neutrophils are the first blood cells to migrate into the injured myocardium. Monocytes are also involved in plaque progression and play a crucial role in myocardial injury resolution and subsequent cardiac remodeling, while lymphocytes are well-known mediators of the immune response and inflammation. Thus, research has increasingly focused on complete blood count (CBC) parameters alone, CBC-derived indices, and other blood cell-related investigations as accessible and reliable biomarkers for CVD. In this section, we discuss recent findings on the use of such blood cell-related parameters as emerging predictive biomarkers for mortality and CV outcomes. Relevant statistical data can be found in Table 3.

#### 3.3.1. CBC Parameters

White blood cells (WBC) and WBC subsets may be associated with risk of HF. A LIPID substudy of 7101 patients who had an ACS 3–36 months before enrollment showed that a higher WBC count predicted the development of HF-related events over a period of follow-up of 5 years [29]. A multicentric study from Turkey, involving 392 patients with HF with reduced ejection fraction (HFrEF) [30], reported that lymphocyte count was lower in patients with NYHA classes III/IV compared to classes I/II: 0.9 [0.6–1.5] vs. 1.5 [0.7–2.2] × 10^3^/µL, *p* < 0.001. In multivariate analysis, decreased lymphocyte count was associated with poor NYHA function independent of CHD risk factors [30]. When looking at monocyte subsets, a Chinese case–control study of 100 patients treated with PCI for de novo STEMI, showed that intermediate monocytes (CD14++CD16+, 2.26-fold increase) and intermediate monocyte-platelet aggregates (MPA, 2.69-fold increase) were elevated during the first 7 days post-STEMI compared to stable CHD controls, with a peak at day two [31]. Out of the three monocyte subsets investigated (classical, intermediate, and non-classical) and their MPAs, intermediate monocytes (CD14++CD16+) and their MPAs were the only predictors for MACE after a 2-year follow-up (CV death, recurrent MI, HF hospitalization, emergency or repeat vascularization, or nonfatal ischemic stroke) [31].

Some platelet and red blood cell parameters were also found to have predictive value for HF and mortality. A Polish retrospective observational study of 278 patients with a one-year follow-up after at least one stent implantation for ACS showed that platelet distribution width (PDW) was independently associated with both systolic HF and one-year mortality [32]. According to ROC analysis, a PDW value > 12.8% could predict the occurrence of 1-year HF with LVEF ≤ 35% after ACS treated with primary PCI (AUC 0.614, 81% sensitivity, 39% specificity, *p* = 0.0177) [32]. A large biobank study of 500,451 individuals from the UK identified mean reticulocyte volume (fL) as a novel biomarker of incident heart failure over a median follow-up of 6.2 years [33]. A study of 443 patients with acute heart failure with preserved ejection fraction (HFpEF) showed that higher red cell distribution width (RDW) and lower mean corpuscular hemoglobin concentration (MCHC) were individually associated with all-cause mortality over a follow-up period of 2.2 years [34].

#### 3.3.2. CBC-Derived Indices

The same study of HFpEF patients mentioned above also reported that neutrophil to lymphocyte ratio (NLR) on admission and absolute NLR trajectory during hospitalization were independently associated with all-cause mortality [34].

A study of 1754 patients from the Utrecht Coronary Biobank aimed to analyze the association of baseline monocyte to lymphocyte ratio (MLR) with baseline characteristics of patients who underwent coronary angiography, as well as baseline MLR association with future HF hospitalization [35]. At baseline, univariate analysis showed that a 1-point increase in logMLR was associated with unstable angina vs. stable CAD, MI vs. stable CAD, and 1-category poorer EF [35]. In multivariate analysis, MLR was associated only with unstable angina vs. stable CAD. Moreover, high MLR predicted HF hospitalization over a follow-up period of 484 days [35]. A combined monocyte to HDL–cholesterol ratio (MHR) was also found to have predictive value in a study of 231 patients admitted with STEMI for the first time, who underwent primary PCI in the first 12 h [36]. MHR at the time of admission was higher in patients with adverse cardiac remodeling at the 6-month follow-up [36]. Although several hematological parameters and indices at admission were significantly associated with adverse cardiac remodeling in univariate analysis, only MHR remained significant in multivariate analysis [36]. MHR was superior to other indices in predicting adverse cardiac remodeling with an established threshold of 1.6: 92.7% sensitivity, 70.1% specificity; AUC 0.84 [0.78–0.88], positive predictive value 46.8%, negative predictive value 96.7%, *p* < 0.001 [36]. Values of MHR >1.6 were also predictive for all-cause mortality [36].

Platelet-related indices may also have predictive value in CHD patients. A study of 5886 patients admitted with STEMI [37] showed that, after a 81.6-month follow-up, adjusted all-cause mortality was higher in patients with a higher platelet to lymphocyte ratio (PLR). In adjusted analysis, a higher PLR quartile (2nd to 4th) was also predictive for recurrent MI, HF, and ischemic stroke [37]. In ROC analysis, a PLR threshold of 132.6 was shown to have 68.5% specificity and 71.3% sensitivity for the prediction of clinical outcomes (AUC = 0.704, *p* < 0.001) [37]. A Chinese retrospective observational cohort on 2599 patients with CAD complicated by congestive heart failure, reported that high platelet to hemoglobin ratio (PHR) was associated with a higher risk of all-cause mortality in unadjusted, age- and gender-adjusted, and fully adjusted models over a median follow-up of 5.2 years [38]. Patients in the upper quartile of PHR were shown to have an increased risk of all-cause mortality compared to those in the lowest quartile [38].

The systemic immune-inflammation index (SII) is an emerging, more complex biomarker that takes into consideration absolute values of three CBC parameters: SII = (neutrophil count × platelet count)/lymphocyte count. A retrospective observational study of 843 patients with STEMI investigated the association of SII with MACEs [39]. Patients were divided in two groups according to the median SII. In the adjusted Cox regression models, patients with high SII were more likely to go through a MACE during the 34.2-month follow-up period [39]. Regarding individual outcomes, higher SII was associated with cardiac death, non-fatal MI, non-fatal stroke, congestive HF requiring hospitalization, and revascularization interventions (PCI or coronary artery bypass graft—CABG) [39]. The ROC analysis revealed that a SII cut-off value of 951.7 could predict MACE with 64.6% sensitivity and 73.6% specificity (AUC 0.741, *p* < 0.0001) [39]. The predictive value of the three-variable SII was superior to its two-variable counterparts, namely NLR and PLR [39].

#### 3.3.3. Blood Cell-Related Parameters Improve Existing Models

In acute HFpEF patients, the addition of both NLR on admission and absolute NLR trajectory (admission to discharge) improved the model combining GWTG-HF (Get With the Guidelines–Heart Failure) score and NT-proBNP as well as GWTG-HF score alone, enhancing its 1-year, 2-year, and 3-year predictive value for mortality [34]. MLR modestly, but significantly, improved risk prediction in patients undergoing coronary angiography when added to the full HF prediction model [35]. A retrospective observational study of 843 patients with STEMI showed that, when added to the clinical model based on traditional risk factors, SII significantly improved the post-STEMI prediction of individual CV outcomes and overall MACE [39].

**Table 3:** Predictive value of hematological biomarkers for clinical outcomes in patients with ischemia-related cardiovascular diseases.

Biomarker	Details	Outcome(s)	Follow-Up	n	Predictor/Cut-Off Value	Risk [95% CI]	Ref.
WBC	Stable CHD (ACS history)	HHF or HF death	5 years	7100	Higher	HR 1.07 [1.02–1.12]	[29]
Lymphocyte count	HF with LVEF ≤ 40%	Poor NYHA functional class	N/A	392	Lower	OR 0.60 [0.37–0.96]	[30]
PDW	ACS + PCI (≥1 stent)	Systolic HF, LVEF ≤35%	In-hospital	278	>12.8%	OR 2.81 [1.14–6.92]	[32] *
		Mortality	1 year		>16%	OR 2.67 [1.01–7.02]	
MRV	UK Biobank study	Incident HF	6.2 years	500,000	Higher	HR 1.19 [1.01–1.39]	[33]
RDW	Hospitalization with acute HFpEF	All-cause mortality	2.2 years	443	Higher	HR 1.20 [1.07–1.37]	[34] *
MCHC					Lower	HR 0.82 [0.68–0.99]	
NLR on admission					Higher	HR 1.20 [1.01–1.40]	
NLR trajectory					Higher	HR 1.22 [1.07–1.41]	
MLR	Undergoing CAG (symptomatic > 50% stenosis, UA, or MI)	HHF	484 days	1754	Q4 (>0.43) vs. Q1–3	HR 2.10 [1.10–4.10]	[35]
MHR	First STEMI + PCI	All-cause mortality	1.5 years	231	>1.6	HR 5.62 [2.01–15.70]	[36]
		Adverse remodeling			Higher	OR 3.21 [1.51–84.00]	
PLR	STEMI	All-cause mortality	81.6 months	5886	Q4 (>163.3) vs. Q1 (<98.8); (data for Q2–3 not shown)	HR 1.59 [1.33–1.94]	[37] *
		Recurrent MI				HR 1.34 [1.27–1.65]	
		HF				HR 1.36 [1.24–1.72]	
		Ischemic stroke				HR 1.29 [1.11–1.46]	
PHR	Undergoing CAG; diagnosis: CAD with CHF	All-cause mortality	5.2 years	2599	≥1.69	HR 1.31 [1.13–1.52]	[38] *
SII	STEMI	MACE: CV death, MI, or stroke	34.2 months	843	≥554.9	HR 8.51 [4.45–16.26]	[39] *
		Cardiac death				HR 3.06 [1.75–5.35]	
		Non-fatal MI				HR 2.78 [1.65–4.68]	
		HHF				HR 11.11 [4.13–29.85]	
		Revascularization (PCI or CABG)				HR 4.11 [1.88–8.96]	

* Retrospective studies. Abbreviations: ACS, acute coronary syndrome; CABG, coronary artery bypass graft; CAD, coronary artery disease; CAG, coronary angiography; CHD, coronary heart disease; CHF, congestive heart failure; CI, confidence interval; CV, cardiovascular; HF, heart failure; HFpEF, heart failure with preserved ejection fraction; HHF, heart failure hospitalization; HR, hazard ratio; LVEF, left ventricular ejection fraction; MACE, major adverse cardiovascular event; MCHC, mean corpuscular hemoglobin concentration; MHR, monocyte/HDL ratio; MI, myocardial infarction; MLR, monocyte-to-lymphocyte ratio; MRV, mean reticulocyte volume; n, number of participants in the study; NLR, neutrophil-to-lymphocyte ratio; NYHA, New York Heart Association (functional classification); OR, odds ratio; PCI, percutaneous coronary intervention; PDW, platelet distribution width; PHR, platelet-to-hemoglobin ratio; PLR, platelet-to-lymphocyte ratio; RDW red cell distribution width; Ref, reference; SII, systemic immune-inflammation index; STEMI, ST segment elevation myocardial infarction; UA, unstable angina; WBC, white blood cell count.

### 3.4. Parameters of the Carbohydrate Metabolism

In clinical settings, the investigation of the carbohydrate metabolism heavily relies on few parameters, namely plasma glucose (fasting, random, or after an oral glucose tolerance test—OGTT) and glycated hemoglobin (HbA1c). Impaired glucose metabolism is a well-established risk factor for CHD and patients with diabetes mellitus are at increased risk of CVD and mortality. Emerging evidence indicates that the parameters of the carbohydrate metabolism may be used to predict CV outcomes after ACS, both in healthy and diabetic patients. In this subchapter, we summarize recent findings regarding the use of glycemia and other biomarkers of carbohydrate metabolism for predicting mortality and CV events in ACS patients. Relevant statistical data can be found in Table 4.

#### 3.4.1. Admission Plasma Glucose (APG) in ACS

Plasma glucose concentration is an inexpensive routine laboratory investigation that is part of virtually every chemistry panel performed in emergency departments. As such, several studies have investigated the association of APG with clinical outcomes in patients with ACS presenting to the emergency department.

APG was shown to be associated with in-hospital outcomes. A study of 667 patients admitted with first STEMI reported that APG ≥11.1 mmol/L was the second-best predictor of in-hospital mortality after cardiac arrest [40]. Similarly, a study of 1168 Black Africans with ACS [41] reported that elevated APG was associated with in-hospital mortality at an even lower threshold (>7.8 mmol/L). However, subgroup analysis revealed that APG predicted in-hospital mortality only in patients without diabetes (*n* = 836) [41]. APG was also shown to be associated with clinical outcomes after patient discharge following ACS [41]. A large study of 5309 STEMI and NSTEMI patients treated with PCI investigated the association of APG with a medium-term composite CV outcome (first of mortality, MI, HF, stroke) within 180 days from admission [42]. In patients without known diabetes, grouped based on APG according to WHO criteria, the incidence of the composite outcome increased with increasing APG group [42]. Higher APG was also predictive for the composite outcome in patients with diabetes [42]. For longer-term prediction of outcomes, a study of 417 patients who were admitted with ACS and treated by PCI reported that APG >10 mmol/L was predictive for MACCEs over a 39-month period (cardiac death, recurrent ACS, revascularized angina, acute decompensated HF, or stroke) [43].

#### 3.4.2. Glycemic Variability in ACS

The study of 417 patients mentioned above also investigated the predictive value of glycemic variability in ACS patients treated by PCI [43]. Using continuous glucose monitoring for at least 24 h, the study showed that the mean amplitude of glycemic excursion (MAGE) was correlated with MACCEs over a mean follow-up period of 39 months [43]. In a multivariate model, including high MAGE, glucose >10 mmol/L, and HbA1c, only MAGE remained predictive for MACCEs. High MAGE remained significantly associated with MACCEs even after multivessel disease, HDL-C, BNP, and hs-CRP were added to the model [43]. Moreover, subgroup analysis revealed that MAGE also remained significantly associated with MACCEs in patients with diabetes or patients with impaired glucose tolerance [43].

#### 3.4.3. Fasting Plasma Glucose (FPG), Impaired Glucose Tolerance (IGT), and HbA1c

Data from the literature are heterogeneous and contradictory regarding the predictive value of glycemic status in patients with CHD. A Japanese study of patients with CAD undergoing PCI investigated the 10-year association of IGT with a composite outcome of CV death, MI, stroke, repeat revascularization, and HF hospitalization [44]. IGT was shown to be a predictor of long-term CV risk, while previously known and newly diagnosed diabetes were not [44]. Contrarily, a study of 374 patients who underwent PCI reported that a known history of diabetes, but not newly diagnosed diabetes or IGT, was associated with revascularization, non-fatal MI, and readmission for HF after a mean follow-up of 35.8 months [45].

To further diversify the findings, a Swedish cohort study of 841 patients admitted with STEMI [46] and followed for a mean period of 4.8 years reported that glycemic status according to OGTT had no predictive value for the composite outcome (first of MI, HF, ischemic stroke, or mortality). However, glycemic status according to HbA1c was predictive for the composite outcome, but only in patients with prediabetes according to ADA criteria [46]. When comparing different cut-off values for glucose and HbA1c, only HbA1c ≥ 39 mmol/mol was predictive for the composite outcome [46]. Given such diverse study findings, the most appropriate glycemic status indicator remains to be established. Some answers come from a Chinese study of 7762 patients with ACS and diabetes that looked at patients with inconsistencies between the two most common parameters used to determine glycemic status, namely FPG and HbA1c [47]. Inconsistencies were defined as either both HbA1c ≥ 48 mmol/mol and FPG < 7.0 mmol/L, or both HbA1c < 48 mmol/mol and FPG ≥ 7.0 mmol/L. The study showed that inconsistencies in FPG and HbA1c levels result in two patient categories with different in-hospital outcomes. After adjusting for confounding factors, patients in the increased FPG group were more likely to suffer adverse CV outcomes compared with those in the increased HbA1c group [47]. Thus, in the setting of ACS, among diabetic patients with HbA1c-FPG discrepancy, those with increased FPG are at a higher risk of in-hospital adverse outcomes compared to those with increased HbA1c [47].

Beyond differences generated by distinct study designs and cohort sizes, study findings remain heterogeneous regarding the predictive value of common glycemic status indicators in CHD patients. Nevertheless, it seems that in the acute setting, glycemic classification based on glycemia tests is superior to that based on HbA1c. Although distinct clinical outcomes are apparent between nondiabetic, prediabetic, and diabetic patients, further research is required, particularly for identifying the best predictive glycemic status indicator in different clinical scenarios.

#### 3.4.4. Advanced Glycation End Products (AGEs)

A study of two age- and gender-matched cohorts of patients with either ACS or HF (*n* = 102 for each) investigated the 5-year predictive role of AGEs and their receptors (RAGE and sRAGE—soluble form) for cardiac death, non-fatal MI, or HF readmission [48]. ROC analysis revealed that fluorescent AGE (AUC 0.703 [0.597–0.809], *p* = 0.001), sRAGE (AUC 0.623 [0.512–0.734], *p* = 0.038) and endogenous secretory (es)RAGE (AUC 0.621 [0.510–0.713], *p* = 0.042), but not cleaved RAGE were predictive for cardiac death in the HF cohort [48]. However, none of these parameters showed significant AUC in the ACS cohort. Similarly, glycated albumin (AUC 0.680 [0.574–0.786], *p* = 0.002) and esRAGE (AUC 0.631 [0.521–0.741], *p* = 0.024) were predictive for reinfarction and HF readmission outcomes in the HF cohort, but not in the ACS cohort [48]. Adjusted regression models showed that the AGE-RAGE axis was an independent predictor of long-term CV events in the HF cohort, the best prognostic factor being esRAGE >435 pg/mL, regardless of the considered endpoint (HR 2.14 [1.11–4.12], *p* = 0.023). The AGE-RAGE axis did not show such predictive value in the ACS cohort [48].

#### 3.4.5. Ketone Bodies (KBs)

Although KBs are more related to the lipid metabolism, they are discussed here due to their pathophysiological relevance in diabetes. Diabetes is the most frequent pathological cause of elevated KBs in blood. A study of 369 participants from GIPS-III trial, with early metformin therapy after STEMI [49], reported that circulating KBs were higher at presentation with STEMI and at 24 h after reperfusion, compared with levels at four-month follow-up (*p* < 0.001). More importantly, increased KBs concentrations at 24 h after reperfusion were independently associated with larger MI size and lower LVEF [49]. The most abundant circulatory KB, β-hydroxybutyrate, was also associated with CV outcomes in a study of 405 stable hemodialysis patients over a mean follow-up of 3.2 years [50]. MACE rates increased with increasing quintiles from 11.1/1000 person-years in the lowest quintile (<89 µmol/L) to 80.1/1000 person-years in the highest quintile (>409 µmol/L). In adjusted analysis, the highest quintile was predictive for MACE when compared to the lowest quintile (cardiac death, nonfatal MI, HF hospitalization, or nonfatal stroke) [50].

**Table 4:** Predictive value of parameters of the carbohydrate metabolism for clinical outcomes in patients with ischemia-related cardiovascular diseases.

Biomarker	Details	Outcome(s)	Follow-up	n	Predictor/Cut-Off Value	Risk [95% CI]	Ref.
APG	STEMI/NSTEMI + PCI; no known DM	MACE: death, MI, HF, or stroke	180 days	5309	7.0–11.0 mmol/L vs. <6.1 mmol/L (126–198 mg/dL vs. <110 mg/dL)	OR 1.62 [1.14–2.29]	[42]
					>11 mmol/L vs. <6.1 mmol/L (>198 mg/dL vs. <110 mg/dL)	OR 3.59 [1.99–6.50]	
	STEMI/NSTEMI + PCI; known DM				Higher	OR 2.42 [1.71–3.42]	
	ACS ± DM (Black Africans)	Mortality	In-hospital	1168	>7.8 mmol/L (>140 mg/dL)	HR 2.33 [1.44–3.77]	[41]
	ACS, no DM (Black Africans)			836		HR 3.12 [1.72–5.68]	
	First STEMI, no PCI	Mortality	In-hospital	667	≥11.1 mmol/L (≥200 mg/dL)	OR 2.62 [1.74–3.93]	[40]
					Higher	HR 3.17 [2.46–4.09]	
	First ACS + PCI; CGM ≥ 24 h	MACCE: cardiac death, recurrent ACS, RA, ADHF, or stroke	39 months	417	>10 mmol/L (>180 mg/dL)	OR 1.72 [0.86–3.34]	[43]
Glycemic variability (MAGE)					Higher	OR 1.84 [1.01–3.35]	
		MACCE; DM patients only			Higher	OR 3.23 [1.04–12.38]	
		MACCE; IGT patients only			Higher	OR 2.08 [0.86–4.95]	
HbA1c		MACCE			Per 1% increase	OR 1.05 [0.80–1.36]	
	MI, no DM	Composite: MI, HF, ischemic stroke, or death	4.8 years	841	39–47 vs. <39 mmol/mol (5.7–6.5% vs. <5.9%)	HR 1.31 [1.05–1.63]	[46]
					≥39 vs. <39 mmol/mol (≥5.7% vs. <5.7%)	HR 1.30 [1.05–1.61]	
Groups of HbA1c and FPG	ACS and DM	MACCE: CV death, CA, CS, recurrent MI, stent thrombosis, or stroke	In-hospital	7762	HbA1c < 48 mmol/mol and FPG ≥ 7 mmol/L vs. HbA1c ≥ 48 mmol/mol and FPG < 7 mmol/L (HbA1c < 6.5% and FPG ≥ 126 mg/dL vs. HbA1c ≥ 6.5% and FPG < 126 mg/dL)	OR 1.52 [0.85–2.72]	[47]
		HF				OR 1.63 [1.07–2.48]	
		CV death or HF				OR 1.63 [1.08–2.47]	
		MACCE or HF				OR 1.57 [1.07–2.28]	

Abbreviations: ACS, acute coronary syndrome; ADHF, acute decompensated heart failure; APG, admission plasma glucose; CA, cardiac arrest; CGM, continuous glucose monitoring; CI, confidence interval; CS, cardiogenic shock; CV, cardiovascular; DM, diabetes mellitus; FPG, fasting plasma glucose; HbA1c, glycated hemoglobin; HF, heart failure; HR, hazard ratio; IGT, impaired glucose tolerance; MACE, major adverse cardiovascular event; MACCE, major adverse cardiac and cerebrovascular events; MAGE, mean amplitude of glycemic excursion; MI, myocardial infarction; n, number of participants in the study; NSTEMI, Non-STEMI; OR, odds ratio; PCI, percutaneous coronary intervention; RA, revascularized angina; Ref, reference; STEMI, ST segment elevation myocardial infarction.

### 3.5. Urinary and Kidney-Related Parameters

Although impaired kidney function can frequently be associated with CV conditions, it is unclear whether renal parameters contribute significantly to risk stratification or adverse outcome prediction. Despite the fact that blood remains the sample of choice for the determination of biomarkers, urinary biomarkers have gained attention owing to the uncomplicated, non-invasive collection technique and reduced costs. This section presents recent findings concerning the predictive value of urinary and kidney-related biomarkers in the setting of CV disease. Relevant statistical data have been included in Table 5.

#### 3.5.1. Serum Creatinine and Blood Urea Nitrogen

The negative prognostic role of acute kidney injury in STEMI patients is undisputed; however, the relationship between subclinical increased serum creatinine levels and adverse cardiac outcomes in this category of patients is still uncertain. A retrospective analysis of 1897 STEMI patients who underwent primary PCI revealed that subclinical acute kidney injury (delineated by increases in serum creatinine of ≥0.1 mg/dL, but <0.3 mg/dL) was independently associated with the composite endpoint (defined as HF, atrial fibrillation, need for mechanical ventilation, and in-hospital mortality) [51]. Another retrospective report focused on blood urea nitrogen (BUN) levels for predicting in-hospital mortality in 2995 patients with AMI [52]. On multivariate analysis, higher levels of BUN had a statistically significant association with in-hospital mortality [52].

A prospective study investigated the prognostic role of the blood urea nitrogen/creatinine ratio (BUN/Cr) in 389 consecutive patients undergoing PCI for MI during a follow-up period of 1 year [53]. The subgroup of patients with acute HF as a complication of MI, and high BUN/Cr (>15.34) had a significantly increased 1-year mortality rate compared to the other enrolled patients. However, there was no significant difference in predictive value for the combination of acute HF and BUN/Cr compared to the established GRACE risk score (ROC analysis AUC = 0.695 vs. 0.707, *p* > 0.05) [53].

#### 3.5.2. Cystatin-C

Cystatin-C is a marker of the renal function independent of age, sex, or muscle mass (contrary to serum creatinine), proposed in multiple recent studies for CV risk prediction. Serum cystatin-C was evaluated in relation to an outcome consisting of CV death or HF hospitalization in a substudy of the SOLID-TIMI 52 trial (*n* = 4965 patients with ACS ≤1 month, followed up for a median of 2.5 years) [54]. Increasing levels of cystatin-C were associated with a higher risk of the primary outcome, a higher risk of CV death alone, and a higher risk of HF hospitalization alone [54]. Adding this biomarker to an adjusted risk model, which excluded estimated glomerular filtration rate (eGFR), did not significantly increase prediction value for the primary outcome (C-statistic 0.81 vs. 0.80, *p* = 0.03) [54].

A retrospective analysis investigated the predictive role of cystatin-C in 277 patients with preserved eGFR (eGFR >60 mL/min/1.73 m^2^) who underwent elective PCI with drug-eluting stents [55]. Patients in the group with high cystatin-C values (>0.637 mg/L) had a significantly higher rate of MACCE (major adverse cardiac and cerebrovascular events, composed of CV death, ACS, stroke, and CHF hospitalization) after a 63-month median follow-up on multivariate analysis [55]. Additionally, a substudy of the EXAMINE trial, involving 5380 patients with recent ACS and type 2 diabetes mellitus, showed that cystatin-C was significantly associated with the composite endpoint (nonfatal MI, nonfatal stroke, or CV death) and mortality in this particular category of patients [56].

#### 3.5.3. Neutrophil Gelatinase-Associated Lipocalin

Neutrophil gelatinase-associated lipocalin (NGAL), a small-size circulating protein first found in activated neutrophils, is a biomarker of renal dysfunction and has been investigated for its potential role in non-renal pathology, including CV disease [57].

A study of 673 patients admitted with STEMI [58] reported that NGAL on admission (before PCI) was predictive for 1-year mortality. However, when BNP was introduced in the model (*n* = 431), NGAL lost its predictive value. C-statistics showed that NGAL ≥84 pg/mL was a decent predictor of 1-year all-cause mortality, acute HF hospitalization, combined death and/or acute HF hospitalization, revascularization, and reinfarction [58]. Moreover, NGAL improved the predictive value of TIMI clinical score regarding 1-year mortality and combined 1-year mortality and/or hospitalization for acute HF. This combined score was superior to the one based on TIMI and BNP and was not improved by the addition of BNP [58].

#### 3.5.4. Urinary Biomarkers

The measurement of biomarkers related to CVD outcomes from urine samples could help to better understand the correlations between renal function and the development of CVD. A report including a very large number of participants (*n* = 478,311) of the UK Biobank revealed an inverse association between the urine sodium–potassium ratio (UNa/UK) from random spot measurements and CAD [59]. A post hoc analysis of the ESPRIT study (*n* = 520 patients) showed that high sodium levels from measurements of spot urine (UNa ≥ 4 g/day) were associated with HF hospitalization, but not with other CV adverse outcomes [60]. On the other hand, as far as the serum measurements of sodium are concerned, a longitudinal observational study of 3558 patients with incident acute MI demonstrated that hyponatremia on admission (Na < 136 mmol/L) was a predictor of mortality [61].

Urinary 8-hydroxydeoxyguanosine (8-OHdG), an indicator of oxidative stress, is a novel and promising urinary biomarker, investigated in a prospective observational study of 515 patients diagnosed with ACS [62]. This study showed the independent predictive value of elevated admission urinary 8-OhdG on MACE (composed of CV death, nonfatal MI, and hospitalization for HF) and CV death alone [62].

**Table 5:** Predictive value of kidney related parameters and urinary markers for clinical outcomes in patients with ischemia-related cardiovascular diseases.

Biomarker	Details	Outcome(s)	Follow-Up	n	Predictor/Cut-Off Value	Risk [95% CI]	Ref.
Creatinine (serum)	PCI for STEMI	HF, AF, need for mechanical ventilation, and in-hospital mortality	In-hospital	1897	0.1–0.3 mg/dL	OR 1.92 [1.23–2.97]	[51]
BUN	MI patients	In-hospital mortality	In-hospital	2995	Tertile 3 (19.5–240 mg/dL) vs. Tertile 1 (1.5–14.4 mg/dL)	OR 2.59 [1.57–4.25]	[52]
BUN/Creatinine	MI + PCI	Mortality	1 year	389	>15.34 + acute HF	HR 5.57 [1.86–16.66]	[53]
Cystatin-C	Recent ACS	CV death or HHF	2.5 years	4965	Per SD increase of log-transformed values	HR 1.28 [1.12–1.46]	[54]
		CV death				HR 1.24 [1.04–1.47]	
		HHF				HR 1.42 [1.19–1.69]	
	Elective PCI + DES (preserved eGFR)	CV death, ACS, stroke, and CHF hospitalization	63 months	277	>0.637 mg/L	HR 1.30 [1.01–1.63]	[55]
	Recent ACS + type 2 DM	Nonfatal MI, nonfatal stroke, or CV death	18 months	5380	Per 1 SD increase	HR 1.28 [1.14–1.45]	[56]
		Mortality				HR 1.51 [1.30–1.74]	
UNa/UK	No baseline CVD	CAD	6.1 years	478,311	Per SD change	HR 0.96 [0.93–0.98]	[59]
UNa (spot)	≥1 diagnosed CV condition	HHF	5.2 years	520	≥4 g/day	HR 1.75 [1.05–2.83]	[60]
Serum Na (admission)	Incident MI	Incident MI	6 years	3558	<136 mmol/L	HR 1.61 [1.32–1.97]	[61]
Urinary 8-OHdG (admission)	ACS	CV death, nonfatal MI, and HHF	34 months	515	≥17.92 ng/mg creatinine	HR 2.15 [1.00–4.63]	[62]
		CV death				HR 7.64 [1.60–36.45]	

Abbreviations: 8-OhdG, Urinary 8-hydroxydeoxyguanosine; ACS, acute coronary syndrome; AF, atrial fibrillation; BUN, Blood Urea Nitrogen; CAD, coronary artery disease; CI, confidence interval; CV, cardiovascular; CVD, cardiovascular disease; DES, drug eluting stent; DM, diabetes mellitus; eGFR, estimated glomerular filtration rate; HF, heart failure; HHF, heart failure hospitalization; HR, hazard ratio; MI, myocardial infarction; n, number of participants; OR, odds ratio; PCI, percutaneous coronary intervention; Ref, reference; SD, standard deviation; STEMI, ST segment elevation myocardial infarction; UK, urinary potassium; UNa, urinary sodium; UNa/UK, urine sodium–potassium ratio.

### 3.6. Hormones and Mineral Metabolism Markers

#### 3.6.1. Cortisol and Aldosterone

Aldosterone is known to contribute to the pathophysiology of HF, and many drugs for HF target the renin–angiotensin–aldosterone system [63]. Elevated cortisol is known to promote the development of risk factors for atherosclerosis, such as truncal obesity, hyperinsulinemia, hyperglycemia, insulin resistance, and dyslipidemia [64]. Despite their recognized involvement in CVD disease, these hormones are not routinely used as markers of prognosis.

Recent data are fairly scarce. A study of 842 patients admitted with acutely decompensated HF and followed for a median of 38 months [65] reported that in patients naïve to mineralocorticoid receptor antagonists (MRA), higher levels of both aldosterone and cortisol were predictive for increased mortality. By contrast, in MRA-treated patients, aldosterone only, but not cortisol, was predictive for increased mortality. Looking at different corticosteroid profiles, the study found that MRA-naïve patients at highest risk were those with high cortisol (higher tertile) and mid-level or high aldosterone. In MRA-treated patients, only one profile was at risk (low cortisol, high aldosterone), and their risk was the highest in the entire cohort: low cortisol (lowest tertile) and high aldosterone (highest tertile) [65].

#### 3.6.2. Thyroid Hormones

The effects of thyroid hormones on the CV system are well known, as are the clinical changes that occur in hypo- or hyperthyroidism [66]. However, thyroid hormones are not currently considered mainstream biomarkers for CVD. As for cortisol and aldosterone, data on the use of thyroid hormones as outcome predictors in patients with CVD are limited.

A study of 642 CAD patients after ACS showed that thyroid parameters measured at admission in a rehabilitation clinic were able to predict mortality over a follow-up period of 118 months [67]. Both fT4, (ln)fT3/fT4, and (ln)rT3 (reverse T3) were associated with all-cause mortality, while only fT4 and the (ln)fT3/fT4 ratio showed significant associations to cardiac-related mortality. Patients with low fT3/fT4 (cut-off < 0.206) had significantly worse outcomes compared to those with higher fT3/fT4. Regarding fT4, higher levels (cut-off 12.54 pg/mL) were associated with worse outcome compared to lower levels [67]. The fT3/fT4 ratio was also shown to be associated with worse outcomes in patients diagnosed with MI with nonobstructive coronary arteries (*n* = 1162) over a median follow-up of 41.7 months [68]. Patients were divided into three groups based on fT3/fT4 tertiles: tertile 1 ≥ 2.64 (*n* = 387), tertile 2 2.28–2.63 (*n* = 386), and tertile 3 < 2.28 (*n* = 389). Patients with lower fT3/fT4 had a higher incidence of MACE (all-cause death, nonfatal MI, revascularization, nonfatal stroke, and hospitalization for unstable angina or HF) (10%, 13.9%, and 18.2%; *p* = 0.005) and composite endpoint of death, reinfarction, revascularization, or stroke (5.4%, 8.5%, and 11.5%; *p* = 0.035) [68]. Compared to higher fT3/fT4 (tertile 1), lower fT3/fT4 was associated with an increased risk of MACE. ROC analysis identified the optimum cut-off for fT3/fT4 as 2.50 (AUC 0.610 [0.550–0.660], *p* < 0.001). A predictive model combining the fT3/fT4 and TIMI score was significantly superior to TIMI or fT3/fT4 alone [68].

#### 3.6.3. Copeptin

Although not a hormone per se, copeptin is a cleavage product of the preprohormone of arginine vasopressin (also known as the antidiuretic hormone) and a surrogate marker of arginine vasopressin. Recently, only small studies reported data on the use of copeptin as a prognostic marker in CVD

For instance, in a study of 79 patients with non ST-segment elevation ACS, copeptin on admission was higher in non-ST-segment elevation MI (NSTEMI) than in unstable angina, and was also positively, albeit weakly to moderately at best, correlated with each of the GRACE (r = 0.43), TIMI (r = 0.55), and SYNTAX (r = 0.65) scores (*p* < 0.001 for all) [69]. The combination of cTnI (cut-off > 0.07 ng/mL) and copeptin (cut-off > 2.34 ng/mL) was superior compared to cTnI alone for identifying patients with NSTEMI (AUC 0.975 [0.944–1.000] vs. 0.888 [0.819–0.956]) with 100% sensitivity (vs. 57%), 93% specificity (vs. 100%), 93.5% positive predictive value (vs. 100%), and 100% negative predictive value (vs. 70%) [69]. Moreover, multivariate analysis showed that admission copeptin, but not cTnI, was an independent predictor of MACE (cardiac death, re-infarction, re-hospitalization for ischemic events, HF, stroke, and target lesion revascularization) [69].

In a DIGAMI substudy, copeptin levels were measured at admission (*n* = 393), on discharge (*n* = 309), and 3 months after MI (*n* = 288) in patients with type 2 diabetes mellitus [70]. Copeptin at different time points was associated with outcomes, but the most consistent association was for admission copeptin, which maintained its predictive value in adjusted models for CV events, for CV death, and for combined non-fatal MI or stroke [70].

In a long-term study comparing diabetics (*n* = 895) to non-diabetics (*n* = 4187), copeptin levels were associated with CV outcomes in diabetics only, over a mean follow-up of 14.4 years [71]. In this study, copeptin was predictive for the composite outcome of CAD, death, or HF, as well as for each individual outcome. Copeptin maintained its predictive value for the composite outcome after adjustment for each of the following factors: diabetes medication, CRP, NT-proBNP, and glomerular filtration rate [71].

In an age- and gender-matched study of 166 patients with AMI without known diabetes, who were followed for 11.6 years [72], copeptin was higher in patients vs. controls (10.5 vs. 5.9 ng/mL, *p* < 0.01), as well as in patients with abnormal glucose tolerance vs. patients with normal glucose tolerance (12.2 vs. 7.9 ng/mL, *p* < 0.01). However, copeptin levels weres not different between controls with or without abnormal glucose tolerance. In diabetics, copeptin levels after AMI predicted MACEs (AMI, stroke, severe congestive HF, or CV death), CV mortality, and overall mortality in unadjusted analysis, while in controls, copeptin predicted MACE. The follow-up was 11.6 years for patients and 10.4 years for controls [72]. The most relevant statistical data from studies concerning hormones as biomarkers are summarized in Table 6.

#### 3.6.4. Fibroblast Growth Factor 23 (FGF-23) and Klotho

Fibroblast growth factor 23 (FGF-23) is a bone-derived hormone involved in the suppression of phosphate reabsorption and the downregulation of 1,25-dihydroxyvitamin D hormone synthesis in the kidney [73]. In order to exert its renal effects and contribute to the development of the cardiorenal syndrome, FGF-23 needs to bind to certain receptors in the presence of a co-factor called Klotho, promoting phosphaturia. FGF-23 may also have direct effects on the heart, in a Klotho-independent interaction [74]. Experimental studies suggested that, in the heart, FGF-23 may contribute to myocardial hypertrophy, endothelial dysfunction and atherosclerosis, and, therefore, may be used as a biomarker of CV disease [73].

Data on FGF-23 and Klotho have also started to emerge from clinical studies. Despite initial enthusiasm, results from recent clinical studies are conflicting and mostly suggest only a modest contribution of FGF-23 in predicting outcomes in CVD.

Data from the CARDIA study collected from 3151 middle-aged adults (mean age 45 ± 4 years) followed for 7.6 years, initially suggested that the C-terminal FGF-23 (cFGF-23) may be useful for predicting incident CVD and mortality [75]. However, the association of cFGF-23 with incident CVD decreased after each adjustment for clinical variables, and there was no significant association with CVD and mortality after full adjustment for confounders (diabetes mellitus, smoking status, physical activity score, BMI, systolic blood pressure, lipids, antihypertensive drug use, and statin use) [75]. cFGF23 was only predictive of a higher risk of hospitalization for HF in some CVD subtypes [75].

By contrast, a secondary analysis of the SOLID-TIMI 52 trial presented a rather more optimistic outlook on FGF-23 as a marker of prognosis [76]. In this study of 4947 patients, FGF-23 levels were measured within 30 days from an ACS and were shown to be associated with CV outcomes over a median follow-up of 2.5 years [76]. The highest FGF-23 levels (4th quartile, >93.53 RU/mL) were associated with CV outcomes: combined CV death or HF hospitalization, CV death, HF hospitalization, all-cause mortality, combined CV death/MI/stroke, stroke, and atrial fibrillation but not MI. Moreover, when added to the clinical base model, FGF-23 provided incremental value for predicting CV death or HF to validated clinical predictors and multiple biomarkers, including eGFR, BNP, hs-CRP, and hs-cTnI [76]. Results were somewhat poorer in women [76].

A smaller study of 88 patients with STEMI who were revascularized by primary PCI, tested correlations between post-ACS FGF-23 levels and left ventricular function at 4 months, as assessed by cardiac magnetic resonance imaging [77]. FGF-23 measured two days after symptom onset was associated with left ventricular remodeling (defined as a ≥20% increase in LV end-diastolic volume)—152.6 [102.5–241.3] vs. 75.8 [58.6–105.4] RU/mL, *p* = 0.002—a correlation which persisted after adjustments for established biomarkers of cardiac stress (NT-proBNP), myocardial necrosis (hs-cTnT), and inflammation (hs-CRP). Moreover, the addition of FGF-23 to the model based on the previously mentioned established biomarkers for LV remodeling, significantly improved its predictive power [77].

Data on FGF-23 and Klotho also emerged from the PEACE trial, which included patients with chronic coronary syndromes and LVEF ≥ 40% and was designed to demonstrate the benefits of trandolapril [78]. A substudy of 3555 patients from the placebo arm investigated the predictive role of FGF-23 and Klotho (FGF23 co-receptor) for CV outcomes over a 4.8-year follow-up [78]. FGF-23 and Klotho levels were categorized by quartiles. By comparison with higher Klotho concentrations (quartile 4, >691.45 pg/mL), low Klotho (quartiles 1–3) was associated with increased risk for combined CV death or HF hospitalization and CV death, but not HF hospitalization. Patients in quartiles 1–3 of Klotho remained at increased risk for combined CV death or HF hospitalization, and CV death alone, even after adjustment for multiple clinical and biomarker variables. When Klotho and FGF-23 were combined for risk stratification, a five-times higher rate of outcomes was observed for patients with low Klotho (quartiles 1–3) and high FGF-23 (quartile 4) as compared with patients with higher Klotho (quartile 4) and lower FGF-23 (quartile 1–3): *n* = 340, 16.9% vs. 3.4%. The risk persisted after multivariate adjustment [78].

#### 3.6.5. Osteonectin and Serum Phosphate

Osteonectin is a matrix cellular protein which is involved in collagen processing after synthesis in the HF myocardium and in modulating cell adhesion, growth factor activity, and cell cycle [79].

The predictive value of osteonectin was recently assessed in a study of 154 patients with ischemic chronic HF who were followed for up to 3 years (median 2.18 years) [79]. In this study, median osteonectin levels were higher in patients who died by comparison with survivors (907.8 [878.0–937.6] ng/mL vs. 670.9 [636.5–705.3] ng/mL, *p* < 0.001). Additionally, osteonectin was an independent predictor of all-cause mortality and death due to chronic HF, as well as HF-related readmission [79]. Moreover, osteonectin ≥ 845.1 ng/mL predicted cumulative CV events with reasonable sensitivity and specificity (79.2% and 84.4%, respectively). The addition of NT-proBNP to osteonectin did not significantly increase the predictive value [79].

Phosphate is a main structural component of nucleic acids, adenosine triphosphate, and the cell membrane and is also involved in cellular signaling and mineral metabolism. Excess phosphate can have deleterious effects on the CV system, promoting endothelial dysfunction, vascular calcification, and myocardial hypertrophy [80]. In clinical settings, data from two Swedish registries, namely the SWEDEHEART registry and the SCREAM project, suggest the unfavorable effect of hyperphosphatemia on CV outcomes [80]. Xu H. et al. identified 2547 patients from the two registries who were admitted with a suspicion of ACS and reported that higher serum phosphate during hospitalization was associated with in-hospital CV outcomes [80]. Patients with serum phosphate ≥ 1.3 mmol/L (75th percentile) exhibited a higher risk of in-hospital mortality, while patients with serum phosphate ≥ 2.1 mmol/L (95th percentile) had an increased risk for in-hospital events (composite of MI reinfarction, cardiogenic shock, resuscitated cardiac arrest, atrial fibrillation, or atrioventricular block) and in-hospital mortality. Elevated serum phosphate was also predictive for 1-year post-discharge CVD events and mortality [80]

The most relevant statistical data from studies concerning markers of the mineral metabolism are summarized in Table 7.

### 3.7. Omics

#### 3.7.1. Transcriptomics

A whole-genome microRNA (miR) sequencing was performed on whole blood from 199 patients with NSTE-ACS [81]. Generalized linear models were used to investigate associations between miRs and 13 high-risk clinical traits. Data analysis identified 205 pairs of significant miR-risk factor associations (*p* < 0.05). There were 43 miRs associated with chronic HF, 32 miRs associated with renal function, and 30 miRs associated with GRACE score [81]. Chronic HF and GRACE score clustered most tightly together, with 14 miRs shared with a matching fold–change direction. Chronic HF was associated with lower levels of miR-126–5p (*p* < 0.0001), miR-142–5p (*p* = 0.0004), miR-3135b (*p* = 0.0006), and miR-144–5p (*p* = 0.0007). GRACE score correlated inversely with miR-3135b (*p* < 0.0001) and directly with miR-28–3p (*p* = 0.0002) [81].

A study of 2763 participants in the Framingham Heart Study investigated possible associations between HF incidence and 398 circulating extracellular RNAs (ex-RNA) from plasma over a median follow-up of 7.7 years [82]. A total of 12 ex-RNAs were associated with LV mass and at least one other echocardiographic phenotype (left atrial size or LV end-diastolic volume), of which three miRs were associated with lower risk of incident HF (about 15% risk reduction/2-fold increase) after adjustment for clinical variables: miR-20a-5p (HR 0.86 [0.73–1.00], *p* = 0.047), miR-17–5p (HR 0.84 [0.72–0.99], *p* = 0.03), and miR-106b-5p (HR 0.85 [0.73–0.99], *p* = 0.04) [82].

A study of 91 patients with ischemic (59.3%) and non-ischemic (40.7%) cardiomyopathy investigated the association of miR-192 with all-cause mortality over a median follow-up period of 59 months [83]. The expression of miR-192 was classified as low (below median) or high (≥median). The expression of miR-192 was associated with mortality in the whole cohort (Kaplan–Meier *p* = 0.03), with a median age at time of death significantly lower (68 years) for patients with high miR-192 compared with the low miR-192 group (78 years) [83]. However, subgroup analysis revealed that this effect was caused by patients with ischemic cardiomyopathy (*p* = 0.003) and there was no relation between miR-192 expression and mortality in the group with non-ischemic cardiomyopathy. In patients with ischemic cardiomyopathy, the median age at time of death was 67 vs. 84 years in groups with high vs. low miR-192 expression, respectively. Moreover, miR-192 remained significantly associated with mortality even after adjusting for NYHA class, BNP concentration, and EF (*p* = 0.014) [83].

#### 3.7.2. Proteomics

A proteomics and single-cell transcriptomics study measured 1305 plasma proteins 1 month post-MI in 181 patients who were later admitted for HF, and data were compared with those from 250 patients who remained event-free over a 4.9-year follow-up period [84]. Plasma proteins correlated with LVEF at 4 months post-MI were then further enriched on a Singapore cohort of 223 patients post-AMI, of which 33 were admitted with HF over a median follow-up of two years. Data were cross-referenced with findings from the single-cell transcriptome of a HF murine model. In the initial cohort, 212 plasma proteins were associated with future HF events, of which 96 were correlated with LVEF at 4 months post-MI. In the second cohort, only 36 proteins were associated with HF after MI, whereas murine single-cell transcriptomes identified 15 protein candidates. The six most enriched proteins common to all three datasets comprised established biomarkers of HF after MI (NT-proBNP and cTnT), as well as emergent biomarkers: latent transforming growth factor-β binding protein-4, angiopoietin-2, follistatin-related protein-3, and thrombospondin-2 [84].

A matched case–control study evaluated the association of proteomic biomarkers with CV outcomes in 455 controls and 485 cases with CAD and HFrEF after an episode of worsening HF [85]. A total of 276 plasma proteins were analyzed, resulting in 49 proteins significantly associated with clinical outcomes. Seven of these proteins had an adjusted false discovery rate < 0.001: BNP, NT-proBNP, FGF23, growth differentiation factor 15 (GDF15), T-cell immunoglobulin, and mucin domain containing 4 (TIMD4), spondin 1 (SPON1), and pulmonary surfactant-associated protein D (PSP-D). Neither of these proteins were associated with individual clinical events (HF hospitalization, sudden cardiac death, and combined MI or stroke) [85]. Therefore, these proteins were considered strong but indiscriminate predictors of diverse CV events. The addition of these biomarkers to a clinical model significantly improved its predictive value [85].

A substudy of the EXAMINE trial on 5131 patients with type 2 diabetes mellitus and recent MI (15–90 days before randomization) investigated specific proteomic signatures for various CV outcomes over a median follow-up of 1.6 years [86]. The study explored the added predictive value of different proteins on top of a well-calibrated clinical model comprising age, sex, diabetes duration, smoking, previous MI, previous HF hospitalization, previous stroke, hypertension, atrial fibrillation, systolic blood pressure, estimated glomerular filtration rate, statin therapy, and study treatment (algoliptin vs. placebo) [86]. Briefly, the clinical model was improved for various CV outcomes as follows: troponin and BNP for CV death alone and combined CV death, MI, or stroke; troponin, BNP, and trail receptor 2 (TRAILR2) for all-cause mortality and combined CV death or HF hospitalization; BNP and galectin 9 (Gal-9) for HF hospitalization; troponin, FGF23, and alpha-1-microglobulin/bikunin precursor (AMBP) for MI; and troponin alone for stroke. The addition of these biomarkers to the clinical model, significantly improved the predictive value for all outcomes, allowing for a better discrimination of low risk from “true” low risk and high risk from “true” high risk [86].

#### 3.7.3. Metabolomics

A post hoc analysis of the CorLipid trial on 316 patients with CAD and diabetes mellitus after coronary intervention for chronic or acute coronary syndrome aimed to identify metabolomic predictors for various CV outcomes over a median follow-up of 2 years [87]. The primary outcome was defined as a composite of MACCE, repeat revascularization, and CV hospitalizations. The study reported that acylcarnitine ratio C4/C18:2 (adjusted HR 1.89 [1.09–3.29], *p* < 0.01) and apolipoprotein B (adjusted HR 1.02 [1.01–1.04], *p* = 0.01) were independent predictors of the primary outcome [87]. Higher levels of acylcarnitine ratio C4/C18:2 (adjusted β 3.02 [0.09–6.06], *p* = 0.04) and ceramide ratio C24:1/C24:0 (adjusted β 7.36 [5.74–20.47], *p* = 0.02) independently predicted a higher complexity of CAD [87].

### 3.8. Other Emerging and Candidate Biomarkers

Several of the studies included in our review address a wide range of emerging biomarkers that do not fit in any of the previous classes. Many of these biomarkers have been investigated by a single study matching our database search methodology. However, some of these candidate biomarkers are even more promising than those emerging biomarkers presented above or those conventional biomarkers we previously discussed in the first part of this work. For practical reasons, these markers will only be mentioned briefly.

#### 3.8.1. Endothelial Function

Baseline and 1-month changes in big endothelin-1 levels were shown to be associated with combined CV death and hospitalization for worsening HF in patients with LV dysfunction after recent MI [88]. Mid-regional pro-adrenomedullin (MR-proADM) is involved in vascular permeability and microcirculation stabilization by regulating the endothelial barrier [89]. MR-proADM was shown to be associated with long-term mortality and HF in patients admitted with STEMI [90] and could be a potentially useful biomarker for discriminating type 1 from type 2 MI on admission [91]. YKL-40 is a mammalian chitinase-like protein considered a marker of endothelial dysfunction and inflammation [92]. Elevated levels of serum YKL-40 were shown to predict long-term MACE in both STEMI patients treated by primary PCI [93] and hypertensive patients [94]. Relevant data can be found in Table 8.

#### 3.8.2. Oxidative Stress and Antioxidant Potential

Superoxide dismutase (SOD), nitrite/nitrate ratio, neopterin, and ferric-reducing ability of plasma were reported as prognostic factors for all-cause mortality and HF hospitalization in patients with STEMI treated by primary PCI [95]. Moreover, SOD and nitrite/nitrate markers added predictive value to the GRACE risk score [95]. Additionally, SOD, nitric oxide (NO), and neopterin were reported to be predictors of acute kidney injury in patients admitted with STEMI and treated with PCI [96]. A decreased level of biological antioxidant potential at 6 months after a PCI-treated STEMI, was shown to be an independent predictor of long-term CV events [97]. The serial monitoring of antioxidant capacity may serve as a predictor of CV outcomes in STEMI patients.

Uric acid is a paradoxical molecule featuring both oxidant and anti-oxidant effects [98]. The correlation between serum uric acid (UA) and adverse CV outcomes has been investigated in multiple studies in recent years. A large study involving 12,677 patients after MI complicated by reduced LV function, HF, or both, revealed that elevated serum UA was associated with poor outcomes [99]. When added to the full model based on clinical and biological variables, serum UA significantly improved the predictive power for CV mortality [99]. Similar findings were reported in a study of 1440 patients undergoing cardiac rehabilitation after myocardial revascularization and/or cardiac valve surgery [100]. Higher UA levels were associated with increased risk for all-cause mortality, CV mortality, and MACCE [100]. Furthermore, for every 1 mg/dL increase in serum UA levels, a 23% and 32% increase was observed in relative risk for all-cause and CV mortality, respectively [100]. Seric UA visit-to-visit variability was also found to have predictive value in a study of 3202 patients with CAD after successful coronary intervention [101]. There was a linear association between high UA variability (measured in SD) and an incidence of adverse clinical outcomes. Compared with the lowest UA-SD quartile, patients in UA-SD quartiles 3 and 4 were at increased risk for MACE, MI, CV death, congestive HF, and total CV events [101]. Relevant data can be found in Table 9.

#### 3.8.3. Enzymes and Other Proteins

Serum-activated aspartic lysosomal endopeptidase cathepsin D (Cathepsin D) levels after primary PCI for STEMI were decreased in patients with post-STEMI new-onset cardiac dysfunction [102]. Lower levels of cathepsin D were associated with MACE at the 6-month follow-up post-STEMI [102]. Patients with higher levels of nardilysin (*n*-arginine dibasic convertase, a type of metalloendopeptidase) at admission for STEMI were shown to be at increased risk for future all-cause mortality [103]. Higher neprilysin (also known as CD10 or common acute lymphoblastic leukemia antigen—CALLA) levels were associated with stunned myocardium early after STEMI, with better improvement of LVEF at follow-up [104]. Serum matrix metalloproteinase 9 (MMP-9) is a candidate biomarker for the early discrimination of MI from unstable angina and a predictor of poor clinical outcomes in patients with ACS [105].

Galectin-3 was shown to be a potential biomarker for the following outcomes: HF in patients with type 2 diabetes mellitus [106], LV remodeling in patients with anterior-wall MI treated by primary PCI [107], and composite CV death and HF hospitalization at 1 year in patients with first-time STEMI treated by primary PCI [21]. Additionally, elevated levels of pre- and post-operative Galectin-3 were associated with increased risk of mortality after CABG [108]. Stanniocalcin-2 and insulin-like growth factor-binding protein 4 (IGFBP-4) were shown to be independent predictors of readmission for HF and all-cause death in patients treated with early reperfusion after STEMI [109]. In heart-transplanted patients, serum levels of donor-specific antibodies were shown to be associated with a higher vasculopathy burden, higher restrictive myocardial damage, and a higher incidence of MACE [110]. Relevant data can be found in Table 10.

#### 3.8.4. Coagulation Proteins and Other Molecules

D-dimer was shown to be associated with an increased incidence of HF after an ACS [29] and its addition to the GRACE score along with NT-proBNP and fibrinogen, significantly improved the predictive value of GRACE for MACE [111]. An ARIC substudy reported that γ’ fibrinogen is unlikely to influence CVD-related events via its prothrombotic properties [112]. Although a modest predictor of CVD death, γ’ fibrinogen added little information to CVD prediction beyond hs-CRP and/or total fibrinogen [112].

Intestinal microbiota-dependent TMAO (trimethylamine N-oxide) is a potential predictor of MACE and all-cause mortality in patients with chronic HF after MI [113]. TMAO, but not its precursors choline and betaine, was also found to be associated with reduced transplant-free survival in patients with chronic HF, but the association was attenuated after adjustment for other covariates [114]. In models adjusted for clinical variables, higher cyclic guanosine monophosphate (cGMP), a second messenger, was associated with long-term incident CV disease: HFpEF, HF, atherosclerotic CVD, and CHD [115]. However, when NT-proBNP was added to the model, cGMP remained an independent predictor only for atherosclerotic CVD and CHD [115]. Lower levels of serum dihomo-gamma-linolenic acid (DGLA) were shown to be associated with higher incidence of all-cause mortality in elderly patients with recent MI [116]. Relevant data can be found in Table 11.

## 4. Discussion

The purpose of this two-part work was to summarize recent findings regarding the use of blood biomarkers for predicting clinical outcomes in patients with CVD, including ACS and ischemia-related HF. In the first part of our work, we addressed the validated biomarkers for CVD and focused on novel ways to use them. In the second part, we approached the non-conventional, emerging biomarkers, and their potential role in predicting outcomes in CVD.

Despite our systematic and exhaustive approach to selecting recent and relevant articles, the current work has several limitations. The literature search was conducted exclusively on the PubMed database. Therefore, it is possible that the literature may hold more data on the prognostic value of the biomarkers discussed in this work. Moreover, some biomarkers presented in articles not indexed by the PubMed database, may have not been included here at all, due to the abovementioned search strategy. In this two-part work, we aimed only to briefly review the literature and create an up-to-date picture of the predictive value of conventional and emerging biomarkers in ischemia-related cardiovascular disease. However, a lack of in-depth discussion concerning the pathophysiological mechanisms behind each biomarker’s predictive role may be considered a limitation of the present work. Although some biomarkers may have deserved a more extensive description of the underlying mechanisms, we approached this only minimally, as the clinical and statistical heterogeneity of the included studies does not allow for a firm verdict regarding the future predictive implications of these emerging biomarkers.

It seems as though, despite extensive research, a novel, perfect biomarker, with high sensitivity and specificity, free of bias, inexpensive, easy to determine, and widely available, is not within reach at this point. Although many humoral parameters have been tested, a definite conclusion cannot be drawn on any of them. In most cases, the studies in which biomarkers were used included a rather low number of patients. Furthermore, the study designs, outcomes followed, and number of patients enrolled, were very different between studies. In some cases, for the same parameter, different laboratory kits were used, which may also contribute to heterogenous results. Standard cut-off values for the emerging biomarkers are not currently established.

To achieve a truly valuable humoral prognostic parameter, further research is needed, and efforts should be focused towards standardization and targeted applicability. This goal is not yet within reach.

## Figures and Tables

**Table 6:** Predictive value of hormonal markers for clinical outcomes in patients with ischemia-related cardiovascular diseases.

Biomarker(s)	Details	Outcome(s)	Follow-Up	n	Predictor/Cut-Off Value	Risk [95% CI]	Ref.
Aldosterone and cortisol	Acutely decompensated HF A. MRA-naïve patients	Mortality	38 months	842	Aldosterone T3 vs. T1	HR 1.51 [1.02–2.24]	[65]
					Cortisol T3 vs. T1	HR 1.94 [1.28–2.93]	
					Cortisol T3 and aldosterone T2	HR 2.42 [1.29–4.53]	
					Cortisol T3 and aldosterone T3	HR 2.11 [1.10–4.05]	
	Acutely decompensated HF B. MRA-treated patients				Aldosterone T3 vs. T1	HR 1.65 [1.01–2.71]	
					Cortisol T1 and aldosteron T3	HR 5.01 [2.22–11.31]	
Thyroid hormones	CAD patients after ACS	All-cause mortality	118 months	642	fT4 levels	HR 1.15 [1.04–1.27]	[67]
					(ln)fT3/fT4	HR 0.08 [0.02–0.32]	
					(ln)rT3	HR 2.15 [1.01–4.58]	
					fT3/fT4 <0.206 vs. >0.206	HR 2.99 [1.43–6.26]	
					fT4 >12.54 vs. <12.54 pg/mL	HR 2.34 [1.05–5.18]	
		Cardiac-related mortality			fT4	HR 1.15 [1.02–1.29]	
					(ln)fT3/fT4	HR 0.10 [0.02–0.55]	
	MINOCA	MACE: all-cause death, nonfatal MI/stroke, revascularization, or hospitalization for UA/HF	41.7 months	1162	fT3/fT4 2.28–2.63 vs. ≥2.64	HR 1.58 [1.05–2.39]	[68]
					fT3/fT4 <2.28 vs. ≥2.64	HR 2.06 [1.17–3.11]	
Copeptin	Non ST-segment elevation ACS	MACE: cardiac death, re-infarction, re- hospitalization for ischemic events, HF, stroke, or target lesion revascularization	1 year (total follow-up)	79	Admission copeptin	OR 0.01 [0.0–0.8]	[69]
	MI +type 2 DM	CV event (CV death/non-fatal MI/atroke)	2.5 years	393	Admission copeptin	HR 1.34 [1.15–1.56]	[70]
		CV death				HR 1.43 [1.16–1.77]	
		Non-fatal MI or stroke				HR 1.25 [1.02–1.54]	
	Diabetic patients	CAD, death, or HF	14.4 years	895	Baseline copeptin	HR 1.32 [1.10–1.58]	[71]
		CAD				HR 1.33 [1.04–1.69]	
		HF				HR 1.62 [1.09–2.41]	
		Death				HR 1.32 [1.04–1.68]	
	AMI, no known DM	AMI, stroke, severe congestive HF, or CV death	11.6 years (patients) 10.4 years (controls)	166	Copeptin levels in patients	HR 1.15 [1.01–1.32]	[72]
					Copeptin levels in controls	HR 1.17 [1.01–1.36]	
		CV mortality			Copeptin levels in patients	HR 1.24 [1.06.1.46]	
		Overall mortality				HR 1.21 [1.05–1.40]	

Abbreviations: ACS, acute coronary syndrome; AMI, acute myocardial infarction; CAD, coronary artery disease; CI, confidence interval; CV, cardiovascular; DM, diabetes mellitus; HF, heart failure; HR, hazard ratio; MACE, major adverse cardiac events; MI, myocardial infarction; MINOCA, myocardial infarction with nonobstructive coronary arteries; MRA, mineralocorticoid receptor antagonists; n, number of participants; OR, odds ratio; Ref, reference; rT3, reverse T3; T, tertile; UA, unstable angina.

**Table 7:** Predictive value of parameters of the mineral metabolism for clinical outcomes in patients with ischemia-related cardiovascular diseases.

Biomarker(s)	Details	Outcome(s)	Follow-Up	n	Predictor/Cut-Off Value	Risk [95% CI]	Ref.
cFGF-23	Middle-aged adults	HHF	7.6 years	3151	Per doubling	HR 1.52 [1.18–1.96]	[75]
FGF-23	Within 30 days of ACS	CV death or HHF	2.5 years	4947	Q4 (>93.53 RU/mL) vs. Q1–3	HR 2.35 [1.82–3.02]	[76]
		CV death				HR 2.53 [1.81–3.52]	
		HHF				HR 2.26 [1.60–3.19]	
		All-cause mortality				HR 2.27 [1.73–2.97]	
		CV death, MI, or stroke				HR 1.42 [1.17–1.71]	
		Stroke				HR 1.96 [1.28–3.01]	
		AF				HR 1.58 [1.12–2.23]	
	STEMI + PCI	LV remodeling	4 months (CMR)	88	>124 RU/mL	OR 14.1 [2.8–70.9]	[77]
FGF-23 and Klotho	Stable ischemic heart disease and LVEF > 40%	CV death or HHF	4.8 years	3555	FGF-23 Q4 vs. Q1–3	HR 1.83 [1.14–2.93]	[78]
					FGF-23 Q4 and Klotho Q1–3 (≤691.45 pg/mL)	HR 3.99 [1.67–9.56]	
					Klotho Q1–3 vs. Q4 (>691.45 pg/mL)	HR 2.62 [1.35–5.08]	
		CV death alone				HR 5.38 [1.60–18.08]	
Osteonectin (baseline)	Ischemic symptomatic moderate-to-severe chronic HF	All-cause mortality	2.18 years	154	>845.15 ng/mL	OR 1.23 [1.10–1.36]	[79]
		Chronic HF-related death				OR 1.46 [1.22–1.80]	
		Readmission				OR 1.92 [1.77–2.45]	
Serum phosphate	Suspected ACS	In-hospital mortality	1 year (total follow-up)	2547	1.3 mmol/L vs. 1.1 mmol/L (75th percentile vs. 50th percentile)	OR 1.36 [1.08–1.71]	[80]
					2.1 mmol/L (95th percentile)	OR 2.86 [1.68–4.86])	
		MI reinfarction, cardiogenic shock, resuscitated cardiac arrest, AF, or AV block				OR 1.98 [1.28–3.06]	
		1-year post-discharge CVD			1.3 mmol/L	OR 1.17 [1.03–1.33]	
					2.1 mmol/L	OR 1.36 [1.01–1.82]	
		1-year post-discharge mortality			1.3 mmol/L	OR 1.24 [1.06–1.45]	
					2.1 mmol/L	OR 1.95 [1.37–2.77]	

Abbreviations: ACS, acute coronary syndrome; AF, atrial fibrillation; AV, atrioventricular; cFGF-23, C-terminal Fibroblast growth factor 23; CMR, cardiac magnetic resonance; CI, confidence interval; CV, cardiovascular; CVD, cardiovascular disease; FGF-23, Fibroblast growth factor 23; HF, heart failure; HHF, heart failure hospitalization; HR, hazard ratio; LV, left ventricular; LVEF, left ventricular ejection fraction; MI, myocardial infarction; n, number of participants; OR, odds ratio; Ref, reference; STEMI, ST segment elevation myocardial infarction; Q, quartile.

**Table 8:** Predictive value of endothelial function-associated molecules for clinical outcomes in patients with ischemia-related cardiovascular diseases.

Biomarker	Details	Outcome(s)	Follow-up	n	Predictor/Cut-Off Value	Risk [95% CI]	Ref.
BigET-1	MI + LVSD ± DM	All-cause death	12.6 months	463	Tertile 3 vs. tertile 1 (baseline: >8.1 vs. ≤6.0 pg/mL; 1-month change: −2.0 vs. 0.5 pg/mL)	Baseline: OR 4.32 [1.55–12.10] 1-month change: OR 2.52 [1.06–5.97]	[88]
		CV death or HHF				Baseline: OR 3.16 [1.38–7.20] 1-month change: OR 2.36 [1.14–4.90]	
	STEMI + PCI	1-year mortality or HHF (acute)	3 years (minimum)	593	>2.6 pg/mL	OR 2.26 [0.96–5.31]	[95]
MR-proADM		Short-term all-cause mortality	Short-term: 30 days; Long-term: 1105 days	1122	Per doubling	HR 2.67 [1.01–7.11]	[90]
		Short-term CV mortality				HR 2.67 [1.01–7.11]	
		Long-term all-cause mortality				HR 3.23 [1.97–5.29]	
		Long-term CV mortality				HR 3.17 [1.56–6.42]	
		HHF				HR 2.71 [1.32–5.58]	
YKL-40		MACE: recurrent MI, HHF, or death	24 months	324	≥126.8 ng/mL	HR 1.65 [1.14–2.39]	[93]
	Hypertension	MACE: new HF, revascularized CAD, or death	7.89 years	135	Higher	HR 1.02 [1.01–1.02]	[94]

Abbreviations: BigET-1, big endothelin-1; CAD, coronary artery disease; CI, confidence interval; CV, cardiovascular; DM, diabetes mellitus; HF, heart failure; HHF, hospitalization for heart failure; HR, hazard ratio; LVSD, left ventricle systolic dysfunction; MACE, major adverse cardiovascular event; MI, myocardial infarction; MR-proADM, mid-regional proadrenomedullin; n, number of participants in the study; OR, odds ratio; PCI, percutaneous coronary intervention; Ref, reference; STEMI, ST segment elevation myocardial infarction. YKL-40 is also known as chitinase-3-like protein 1.

**Table 9:** Predictive value of oxidative stress-associated molecules for clinical outcomes in patients with ischemia-related cardiovascular diseases.

Biomarker	Details	Outcome(s)	Follow-Up	n	Predictor/Cut-Off Value	Risk [95% CI]	Ref.
Neopterin	STEMI + PCI	1-year mortality or HHF (acute)	3 years (minimum)	593	>9.0 nmol/L	OR 3.21 [1.35–7.61]	[95]
		AKI	In-hospital	427	≥11.4 nmol/L	OR 2.83 [1.15–6.96]	[96]
NOx		1-year mortality or HHF (acute)	3 years (minimum)	593	≥45.0 µmol/L	OR 8.03 [3.12–20.64]	[95]
					>31.5 µmol/L	OR 3.20 [1.42–7.19]	
SOD					>77.5 µg/L	OR 6.98 [2.56–19.04]	
FRAP					>1.1 µmol/L	OR 5.64 [2.23–14.26]	
BAP	STEMI + PCI (serial follow-up measurements)	Death, nonfatal MI/stroke, HHF, revascularization, or UA	24 months	69	6-month BAP ≤2718 µmol/L	HR 2.45 [1.10–5.78]	[97]
Serum uric acid	MI + reduced LVEF/HF/both	All-cause mortality	23 months	12,677	Q4 vs. Q1 (≥420 vs. ≤280 µmol/L or ≥7.0 vs. ≤4.7 mg/dL)	HR 1.36 [1.11–1.67]	[99]
		CV mortality				HR 1.47 [1.17–1.83]	
		HHF				HR 1.28 [1.14–1.43]	
	Post-myocardial revascularization and/or cardiac valve surgery	All-cause mortality	50 months	1440	≥360 µmol/L (≥6.0 mg/dL)	HR 2.00 [1.40–2.90]	[100]
		CV mortality				HR 2.00 [1.20–3.20]	
		MACCE: CV death, nonfatal ACS, HF, or stroke				HR 1.40 [1.00–1.90]	
Serum uric acid variability	Symptomatic CAD + PCI	MACE: CV death, or nonfatal MI/stroke	65.6 months	3202	SD Q4 vs. SD Q1	HR 2.53 [1.78–3.59]	[101]
		MI				HR 2.43 [1.53–3.86]	
		CV death				HR 6.45 [2.52–16.55]	
		Congestive HHF				HR 3.43 [2.32–5.05]	
		Total major CV events				HR 2.72 [2.09–3.56]	

Abbreviations: ACS, acute coronary syndrome; AKI, acute kidney injury; BAP, biological antioxidant potential; CAD, coronary artery disease; CI, confidence interval; CV, cardiovascular; FRAP, ferric-reducing ability of plasma; HF, heart failure; HHF, heart failure hospitalization; HR, hazard ratio; LVEF, left ventricular ejection fraction; MACE, major adverse cardiovascular event; MACCE, major adverse cardiac and cerebrovascular event; MI, myocardial infarction; n, number of participants in the study; NOx, nitrite/nitrate; OR, odds ratio; PCI, percutaneous coronary intervention; Q, quartile; Ref, reference; SD, standard deviation; SOD, superoxide dismutase; STEMI, ST segment elevation myocardial infarction; UA, unstable angina.

**Table 10:** Predictive value of plasma enzymes and other proteins for clinical outcomes in patients with ischemia-related cardiovascular diseases.

Biomarker	Details	Outcome(s)	Follow-Up	n	Predictor/Cut-Off Value	Risk [95% CI]/*p* Value	Ref.
Cathepsin D	STEMI + PCI	LVEF ≤ 35%	6 months	88	≤24.95 RFU (at 6 months)	OR 1.31 [1.09–1.57]	[102]
Nardilysin	STEMI	All-cause death	4.6 years	396	Q4 vs. Q1–3 (>2041 vs. <2041 pg/mL)	HR 3.97 [1.64–9.57]	[103]
Neprilysin (admission)	STEMI + PCI	Lower LVEF	In-hospital	68	>450 vs. <450 pg/mL	*p* < 0.01	[104]
		LVEF increase	7 months			*p* = 0.022	
MMP-9	ACS	Recurrent ischemia, HF, or sudden death	6 months	75	5500 vs. 3000 pg/mL	*p* < 0.001	[105]
log_2_(Gal-3)	DM with recent ACS	CV death, HHF, initiation of loop diuretics, or elevated NT-proBNP at follow-up	18 months	5154	Higher	HR 1.21 [1.03–1.41]	[106]
Gal-3	First STEMI (anterior) + PCI	Left ventricular remodeling	6 months	99	Per 1 ng/mL increase	OR 1.22 [1.06–1.42]	[107]
	First STEMI + PCI	CV death or HHF	1 year	104	≥9.57 ng/mL	HR 8.65 [1.45–51.70]	[21]
	Isolated CABG	All-cause death		1560	Q4 vs. Q1 (pre-operative: ≥11.1 vs. ≤5.4 ng/mL; post-operative: ≥12.1 vs. ≤5.7 ng/mL)	Preop. HR 2.22 [1.40–3.54]; Postop. HR 1.66 [1.16–2.37]	[108]
STC-2	STEMI + PCI	HHF or all-cause death	3.3 years	1085	Per 1 SD increment of natural log-transformed values	HR 2.06 [1.13–3.75]	[109]
		All-cause death				HR 2.23 [1.16–4.29]	
		HHF				HR 3.42 [1.22–9.60]	
IGFBP-4		HHF or all-cause death				HR 1.73 [1.14–2.64]	
DSA	Median 7.5 years after HTx	MACE: significant CAV progression, HF, treated rejection, or CV death	649 days	79	Positive vs. negative	HR 2.90 [1.10–7.20]	[110]

Abbreviations: ACS, acute coronary syndrome; CABG, coronary artery bypass graft; CAD, coronary artery disease; CAV, cardiac allograft vasculopathy; CI, confidence interval; CV, cardiovascular; DM, diabetes mellitus; DSA, donor-specific HLA antibodies; Gal-3, galectin-3; HF, heart failure; HHF, heart failure hospitalization; HR, hazard ratio; HTx, heart transplantation; IGFBP-4, insulin-like growth factor-binding protein-4; LVEF, left ventricular ejection fraction; MACE, major adverse cardiovascular event; MI, myocardial infarction; MMP-9, matrix metalloproteinase-9; n, number of participants in the study; NT-proBNP, N-terminal prohormone of brain (B-type) natriuretic peptide; OR odds ratio; PCI, percutaneous coronary intervention; Q, quartile; Ref, reference; RFU, relative fluorescence unit; SD, standard deviation; STC-2, stanniocalcin-2; STEMI, ST segment elevation myocardial infarction.

**Table 11:** Predictive value of coagulation proteins and other molecules for clinical outcomes in patients with ischemia-related cardiovascular diseases.

Biomarker	Details	Outcome(s)	Follow-Up	n	Predictor/Cut-Off Value	Risk [95% CI]	Ref.
D-dimer	Stable CHD (ACS history)	HHF or HF death	5 years	1729	Q4 vs. Q1 (>1494.9 vs. <613.3 nmol/L)	HR 1.53 [1.17–2.01]	[29]
γ’ fibrinogen	Free of CVD (ARIC study)	CVD death	20 years	10,601	Q4 vs. Q1 (≥35.19 vs. ≤24.34 mg/dL)	HR 1.36 [1.05–1.75]	[112]
					Per 1 SD increase (8.80 mg/dL)	HR 1.10 [1.01–1.20]	
TMAO (plasma)	Chronic HF after MI	MACE: recurrent MI, HHF, or death	672 days	1208	Q4 vs. Q1 (>7.92 vs. <2.83 µmol/L)	HR 1.57 [1.08–2.64]	[113]
		All-cause death				HR 1.53 [1.06–2.51]	
	Stable HF (NYHA II-IV)	All-cause death or HTx	5.2 years	155	Tertile 3 vs. tertiles 1–2	HR 1.79 [0.90–1.79]	[114]
cGMP	Free of HF (ARIC study)	HFpEF	9.9 years	875	Tertile 3 vs. tertile 1 (>4.0 vs. <2.6 pmol/mL)	HR 1.30 [0.79–2.14]	[115]
		Any HF				HR 1.68 [0.88–3.22]	
		ASCVD				HR 2.56 [1.26–5.20]	
		CHD				HR 2.25 [1.07–4.71]	
DGLA	Elderly patients 2–8 weeks after MI	MACE	2 years	1002	Q2–4 vs. Q1 (≥2.44 vs. <2.44%wt)	HR 0.75 [0.55–1.02]	[116]
		Mortality				HR 0.47 [0.26–0.84]	

Abbreviations: ACS, acute coronary syndrome; ARIC, Atherosclerosis Risk in Communities (study); ASCVD, atherosclerotic cardiovascular disease; CAD, coronary artery disease; cGMP, cyclic guanosine monophosphate; CHD, coronary heart disease; CI, confidence interval; CV, cardiovascular; CVD, cardiovascular disease; DGLA, dihomo-γ-linolenic acid; HF, heart failure; HfpEF, heart failure with preserved ejection fraction; HHF, heart failure hospitalization; HR, hazard ratio; HTx, heart transplantation; MACE, major adverse cardiovascular event; MI, myocardial infarction; n, number of participants in the study; NYHA, New York Heart Association (functional classification); OR, odds ratio; Ref, reference; SD, standard deviation; TMAO, trimethylamine N-oxide; Q, quartile; %wt, percent weight of total fatty acids.

## Data Availability

Not applicable.
